# Which evolutionary game-theoretic model best captures NSCLC dynamics?

**DOI:** 10.1371/journal.pone.0347657

**Published:** 2026-06-01

**Authors:** Hasti Garjani, Johan Dubbeldam, Kateřina Staňková, Joel S. Brown

**Affiliations:** 1 Delft Institute of Applied Mathematics, Delft University of Technology, Delft, The Netherlands; 2 Institute for Health Systems Science, Delft University of Technology, Delft, The Netherlands; 3 Integrated Mathematical Oncology, H. Lee Moffitt Cancer and Research Institute, Tampa, Florida, United States of America; Texas Christian University, UNITED STATES OF AMERICA

## Abstract

Understanding and predicting the eco-evolutionary dynamics of cancer requires identifying mathematical models that best capture tumor growth and treatment response. In this study, we fit a family of two-population models to *in-vitro* data from non-small cell lung cancer (NSCLC), tracking drug-sensitive and drug-resistant cells under varying environmental conditions. The dataset, originally presented by Kaznatcheev et al., includes conditions with and without the drug Alectinib and cancer-associated fibroblasts (CAFs). We compare combinations of growth models (logistic, Gompertz, and von Bertalanffy) and drug efficacy terms (Norton–Simon, linear, and ratio-dependent) to identify which best explains the observed dynamics. Our models incorporate density dependence, frequency-dependent competition, and drug response, enabling mechanistic interpretation of tumor cell interactions. The logistic model with ratio-dependent drug efficacy best fits monoculture data. Using growth parameters from monocultures, we estimate inter-type competition coefficients in co-cultures. We find that growth rate and carrying capacity are stable across CAF conditions, while competition and drug efficacy parameters shift, altering interaction dynamics. Notably, CAFs promote coexistence between resistant and sensitive cells, whereas Alectinib results in competitive exclusion. Our results underscore the need to evaluate both model fit and biological plausibility to guide therapeutic modeling of cancer.

## Introduction

Lung cancer is one of the most common cancers diagnosed [1], and it is the leading cause of cancer death [2]. Despite advances in treating cancer, cure for metastatic cancers is rare [3–5]. Mathematical oncology provides mathematical models that can help us understand the response of cancer cells to therapy and improve anti-cancer therapies [6,7].

Increasingly, cancer progression and growth are viewed as an evolutionary game where the best strategy (heritable trait) of an individual cancer cell depends upon the strategies of its neighboring cancer cells and the therapy [8–11]. We can also assume cancer is involved in a leader-follower (Stackelberg) evolutionary game where the physician (leader) applies therapy to which the cancer cells (followers) respond [12–14]. Cancer cells’ interactions with their environment include competition for resources, co-opting of normal cells, responding to immune cells, and engaging in public goods games through angiogenesis and ecosystem engineering [15–17].

Competition within evolutionary games can be driven by three types of processes [18,19]. First, there can be density-independent processes where a cancer cell’s survival and proliferation rates are (nearly) independent of other cells – this is typical of population growth at low population sizes when space and nutrients are abundant. Second, there can be density-dependent processes. These occur when a cancer cell suffers lower fitness (prospects for survival and proliferation) as the density of cancer cells increases – typically occurring through lack of space or nutrients or build-up of toxins. Third, there are frequency-dependent interactions which occur when the fitness of a cell depends on its trait and the frequency of traits among the other cancer cells [20].

In the oncologic literature, there is growing interest in competition assays and experiments where two (or sometimes more) cell lines are followed in mono- and co-cultures to see how the populations grow, interact, and do or do not coexist. Such experiments draw from a long tradition in ecology of competing single-celled protists such as Paramecium or yeast. This tradition goes back to the seminal experiments of Gause in the 1920s and 30s [21]. For cancer, such experiments can reveal the cost of resistance (competing a sensitive and resistant cell line under the presence or absence of drug therapy) [22], the role of glycolysis (competing highly glycolytic cell lines against highly oxidative phosphorylation cell lines) [23], effects of immune cells or fibroblasts (competing cell lines thought to differ in the costs and benefits associated with normal cells), effects of microenvironmental conditions (competing cells lines with differing responses to pH, nutrient deprivation, growth factors, etc.) [24–26], effects of different therapies singly or in combination (competing cell lines through having differing responses), and effects of gene knock-out experiments to discern metabolic pathways (competing knock-out cell lines against their original cell line) [27].

Considering the interactions between the cells as a game, there are roughly three ways to model the trajectory and outcome of competition experiments involving two cell lines. The simplest is to focus entirely on frequency-dependent interactions by using a 2 × 2 matrix game where the cell types represent two strategies, and they experience payoffs (fitness) associated with intra-cell line and inter-cell line interactions. The outcomes can be determined by estimating entries of the payoff matrix and using the replicator equation to derive dynamics towards an equilibrium [28]. This approach subsumes any density-independent and density-dependent processes into the 4 elements of the payoff matrix. A more sophisticated version of the 2 × 2 matrix game occurs when the game is no longer bilinear, meaning that the success of an individual is no longer a linear weighted averaging of payoffs based cell line frequencies. Rather, payoffs can be based on non-linear (concave or convex) combinations of cell-type frequencies [29]. To model the trajectory and outcomes, one requires fitness functions based on these non-linear relationships that can be used to directly derive frequency dynamics. Such a frequency-dependent model will produce trajectories that deviate somewhat from the replicator equation based on the degree of non-linearities. A third approach uses ecological models of competition, such as Lotka-Volterra (L-V) competition equations. Such a model can include density-independent growth, ρ, density dependence via a carrying capacity, *K*, and frequency-dependent interactions via the inter-type competition coefficients, $\alpha_{ij}$. The outcomes of and trajectories of such a model, like the L-V competition equations, are determined by these parameters. Properly fitting such a model requires a relatively large number of treatment combinations. Namely, co-cultures seeded at different frequencies of cell types and ideally at different total initial numbers of cells.

Here, we test for frequency- and density-dependent interactions between two cancer cell lines using data from [25]. They measured the population of two cell lines using 12 initial ratios from zero to one, under four environmental conditions: all combinations of the presence and absence of cancer-associated fibroblasts (CAFs), and the presence and absence of the drug. In [25], they fit their data to the replicator equation, which only considers the frequency dynamics of the two cell types, not the dynamics of their population sizes.

Here, we expand their analyses by explicitly modeling the population dynamics. First, we fit the data to three models of population growth (Logistic, Gompertz, and von Bertalanffy [30,31]) that are commonly applied to cancer. We extend the von Bertalanffy growth dynamics to consider competition between the two cell types in a manner similar to Logistic and Gompertz [32,33]. Second, we compare three ways of including drug efficacy [34]: Norton-Simon model [35], density-independent mortality [36], and density-dependent mortality [37]. Third, we test how intrinsic growth rates (ρ’s as density-independent effects), carrying capacities (*K*’s as density-dependent effects), and competition coefficients ($\alpha_{ij}$’s as frequency-dependent effects) vary with cell type, presence and absence of CAFs, and presence and absence of drug. Finally, we compare our model predictions for the outcomes of competition under the four environments to those of [25].

## Methods

Kaznatcheev et al. [25] conducted competition experiments in cell culture using two non-small cell lung cancer cell lines: one sensitive (parental) and the other resistant to the drug Alectinib. Experimental treatments consisted of all combinations of the presence or absence of cancer-associated fibroblasts (CAFs) and the presence or absence of Alectinib, and 8 seeding frequencies (0, 0.1, 0.2, 0.4, 0.6, 0.8, 0.9, and 1 ratios of sensitive to resistant cells). Each combination was replicated 6 times. To measure population dynamics, sensitive and resistant cell lines were labeled with green fluorescent protein and mCherry fluorescent proteins, respectively. Population sizes as measured by fluorescence intensity were recorded at intervals of 4 hours from time 0–120 hours. When present, Alectinib was introduced into wells as a single dose after 20 hours. Kaznatcheev et al. [25], in evaluating the outcomes of competition, only considered the frequency-dynamics of cells within the wells, namely the ratio of sensitive cells to total population size. In what follows, we use their entire time series of cancer cells’ population sizes to fit models from population ecology. DMSO (dimethyl sulfoxide) is a standard solvent and cryoprotectant added to cell culture growth media. Here, the DMSO environment refers to an environment where CAFs and Alectinib are absent. The other three environments consist of DMSO and combinations of CAFs and/or Alectinib. We investigate models that consider density independence, density dependence, and frequency dependence. We first evaluate how well the data fit three commonly used growth models of population dynamics, adjusted for two-species competitive interactions. Next, we determine the representative drug efficacy model. Then, focusing on the Lotka-Volterra competition models, we test how cell lines, fibroblasts, and the drug influence intrinsic growth rates, carrying capacities, and competition coefficients. Finally, we assess how these parameter variations impact interactions between the different cell types.

### Logistic, Gompertz, and von Bertalanffy growth models

We examined two-population extensions of the Logistic, Gompertz, and von Bertalanffy models. The two population models with Logistic and Gompertz growth have been explored for cancer population growth in the literature [20,33]. The general forms of these two-population models with Norton-Simon drug effects are presented in Eq 1 and 2, respectively.


$$\begin{array}{cc} \dot{S}(t) & =\rho_{1}(1-\frac{S(t)+\alpha_{SR}R(t)}{K_{1}})(1-\lambda C(t))S(t)\\ \dot{R}(t) & =\rho_{2}(1-\frac{\alpha_{RS}S(t)+R(t)}{K_{2}})R(t). \end{array}$$ (1)

$$\begin{array}{cc} \dot{S}(t) & =\rho_{1}\ln(\frac{K_{1}}{S(t)+\alpha_{SR}R(t)})(1-\lambda C(t))S(t)\\ \dot{R}(t) & =\rho_{2}\ln(\frac{K_{2}}{\alpha_{RS}S(t)+R(t)})R(t). \end{array}$$ (2)

The three variables *S*(*t*), *R*(*t*), and *C*(*t*) show the sensitive to drug population, the resistant to drug population, and the amount of drug. We extend von Bertalanffy growth dynamics [30] to a two-population model in Eq 3.


$$\begin{array}{cc} \dot{S}(t) & =\rho_{1}(1-\frac{\sqrt[{3}]{S(t)+\alpha_{SR}R(t)}}{K_{1}})(1-\lambda C(t))S^{\frac{2}{3}}(t)\\ \dot{R}(t) & =\rho_{2}(1-\frac{\sqrt[{3}]{\alpha_{RS}S(t)+R(t)}}{K_{2}})R^{\frac{2}{3}}(t) \end{array}$$ (3)

These models consider density independence through $\rho_{1}$ and $\rho_{2}$, density dependence through *K*_1_ and *K*_2_, frequency dependence through $\alpha_{SR}$ and $\alpha_{RS}$, and drug efficacy through λ. We fit the data to the dataset derived from the *in-vitro* experiment.

We fit each model to each replicate of the experiment individually to examine variability in parameter estimates. The model simultaneously predicts values for resistant and sensitive cells, and given that, in most instances, one population type significantly outnumbers the other, we use a weighted sum of squared errors. The corresponding cost function for each well is denoted as:


$$\begin{array}{c} J_{w}=\sum_{i\in \{S,R\}}\sum_{t=12}^{116}(\frac{d_{it}^{measured}-d_{it}^{model}}{\max_{t}(d_{i}^{measured})}{)}^{2}. \end{array}$$ (4)

where i∈{S,R} denotes the population type, and t∈{12,16,...,116} are the time points in the series. The equation $\frac{d_{it}^{measured}-d_{it}^{model}}{\max_{t}(d_{it}^{measured})}$ presents the error value for cells of type *i* at time *t*. We start from *t* = 12 hour to let the cells settle in the well as suggested by [25]. The $d_{it}^{measured}$ is the measured popula*t*ion at time *t* for population type *i*, $d_{i}^{measured}$ is the vector of the measured populations of type *i* from time 12–116 (measured every four hours), and $d_{it}^{model}$ is the predicted population size using the proposed model.

We use the Chi-square error ($\chi^{2}=J_{w}$) and AIC ($N\ln(\frac{\chi^{2}}{N})+2N_{varys}$ where $N_{varys}$ is the number of variables, and *N* is the number of data points) as our goodness of fit measures in the different wells.

We estimate the model parameters by minimizing the cost function Eq 4, considering the difference between the predicted data points from time 12 hours until time 116 hours and the measured data points. To fit the data to the two-population models, the initial values for the resistant and sensitive populations are set to the measured data values at 12 hours, as Kaznatcheev et al. recommended in their paper. Here, we fit the observations in each well to the model separately to ensure that the intra-subject variations are not averaged out. This helps in assessing whether significant variations occur because of differing experimental conditions, such as varying initial ratios or initial populations.

These models include a maximum of seven different parameters ($\rho_{1}$, $\rho_{2}$, $K_{1}$, $K_{2}$, $\alpha_{SR}$, $\alpha_{RS}$, and λ). First, we fit the data to a model with two unknown parameters (same growth rates and carrying capacities for both cancer cell types) in cases where the drug is not present and a model with three unknown parameters (same growth rates and carrying capacities for both cancer cell types and drug efficacy for the sensitive population) when the drug is present. We increase the number of unknown parameters to at most 5, as seen in Table 1. To prevent overfitting, we increase the number of parameters gradually, considering equal $\rho_{1}$ and $\rho_{2}$ values, equal $K_{1}$ and $K_{2}$values, and $\alpha_{SR}=\alpha_{RS}=1$. We assume distinct growth rates for the two populations $\rho_{1}\neq \rho_{2}$ for the second fit (Logistic 2 of Table 1). For the third and fourth fits, we assume that the model has the same growth rates and one competition coefficient equal to one and an unknown $\alpha_{SR}$ or $\alpha_{RS}$, respectively. Finally, we fit the model with distinct competition coefficients and the same growth rates and carrying capacities for both populations. The models are illustrated in Table 1.

**Table 1:** Models inspired by Logistic growth.

Logistic 1	$\dot{S}(t)=\rho (1-\frac{S(t)+R(t)}{K})(1-\lambda C(t))S(t)$
	$\dot{R}(t)=\rho (1-\frac{S(t)+R(t)}{K})R(t).$
Logistic 2	$\dot{S}(t)=\rho_{1}(1-\frac{S(t)+R(t)}{K})(1-\lambda C(t))S(t)$
	$\dot{R}(t)=\rho_{2}(1-\frac{S(t)+R(t)}{K})R(t).$
Logistic 3	$\dot{S}(t)=\rho (1-\frac{S(t)+\alpha_{SR}R(t)}{K})(1-\lambda C(t))S(t)$
	$\dot{R}(t)=\rho (1-\frac{S(t)+R(t)}{K})R(t).$
Logistic 4	$\dot{S}(t)=\rho (1-\frac{S(t)+R(t)}{K})(1-\lambda C(t))S(t)$
	$\dot{R}(t)=\rho (1-\frac{\alpha_{RS}S(t)+R(t)}{K})R(t).$
Logistic 5	$\dot{S}(t)=\rho (1-\frac{S(t)+\alpha_{SR}R(t)}{K})(1-\lambda C(t))S(t)$
	$\dot{R}(t)=\rho (1-\frac{\alpha_{RS}S(t)+R(t)}{K})R(t).$

Sensitive and resistant population growth follows the ODE model presented at each block. The number of unknown parameters in the Logistic 1 model are two or three depending on whether the drug is present or not (ρ, *K*, and λ). Unknown parameters in Logistic 2 are $\rho_{1}$, $\rho_{2}$, *K*, and λ. Unknown parameters in Logistic 3 are ρ, *K*, $\alpha_{SR}$, and λ. Unknown parameters in Logistic 4 are ρ, *K*, $\alpha_{RS}$, and λ. Unknown parameters in Logistic 5 are ρ, *K*, $\alpha_{SR}$, $\alpha_{RS}$, and λ.

We repeat the same stepwise increase in the number of unknown parameters for Eqs 2 and 3. The tables containing these models are in the S1 Appendix (Table 1 and 2). In total, we fit 36 wells to 15 different models. We compare the error and AIC distribution of the different models to determine the appropriate models.

### Drug efficacy in the two-population model

In addition to growth dynamics, we analyze three models of drug efficacy: the Norton-Simon model, density-independent mortality, and density-dependent mortality. The three mentioned drug effects for the logistic model are illustrated in Eqs 5–7.

1. Norton-Simon model:
  $$\begin{array}{cc} \dot{S}(t) & =\rho_{1}(1-\frac{S(t)}{K_{1}})(1-\lambda C(t))S(t) \end{array}$$ (5)
2. Linear drug effect:
  $$\begin{array}{cc} \dot{S}(t) & =\rho_{1}(1-\frac{S(t)}{K_{1}})S(t)-\lambda C(t) \end{array}$$ (6)
3. Ratio-dependent drug effect:
  $$\begin{array}{cc} \dot{S}(t) & =\rho_{1}(1-\frac{S(t)}{K_{1}})S(t)-\lambda C(t)S(t) \end{array}$$ (7)

We determine the best drug efficacy model by fitting it to the monoculture data. Evidence suggests the model’s structural characteristics (e.g., carrying capacity values, the representative growth model, or drug efficacy) for monoculture and co-culture are similar [38]. Therefore, we use the parameter estimates of the monoculture to develop the two-population model.

### Impact of cell type and the presence or absence of CAFs and drug on model parameters

By fitting data from each well separately, we get replicate parameter estimates, which allow us to compare within-category variance and between-category variance. We used ANOVA (Analysis of Variance) to test the impact of CAFs and therapy on growth rates (density-independent parameters ρ) and carrying capacities (density-dependent parameters *K*).

First, we find the growth rate and carrying capacity of monoculture sensitive and resistant cells in DMSO and CAF environments. Next, we use a two-way ANOVA test for the effects of population type (sensitive and resistant), environment (DMSO and CAF), and their interaction. Then, we use these estimates to estimate the drug efficacy term when the drug is present. The fitted one-population model of sensitive cells:


$$\begin{array}{c} \dot{S}(t)=\rho_{1}S(t)\;(1-\frac{S(t)}{K_{1}})-\lambda S(t)C(t) \end{array}$$ (8)

The fitted one-population model of resistant cells:


$$\begin{array}{c} \dot{R}(t)=\rho_{2}R(t)\;(1-\frac{R(t)}{K_{2}}) \end{array}$$ (9)

The co-culture experiment was also carried out in four distinct environments. We use the growth rate, carrying capacity, and drug effect parameters derived from the monoculture experiment to find the competition coefficients in co-culture. This stepwise approach leverages our prior knowledge of the model in monocultures. The competition coefficients are the only variables that change while fitting the co-culture data.

### Effect of the environment on the interactions among the cells

The experiment was conducted under four environmental conditions, accounting for the presence or absence of CAFs and the presence or absence of the drug. Kaznatcheev et al. [25] only investigated frequency, not density dependent dynamics. They used the replicator equation to forecast outcomes. They predicted the occurrence of a mixed equilibrium (where both sensitive and resistant cells coexist) only in an environment with the presence of CAF, while anticipating the extinction of sensitive cells in all other environments. We fit the data to a model that incorporates density dependence, density independence, and frequency dependence. We examine the influence of the environment on each parameter based on the values of the fitted parameters to gain more insight into the effect of the environment on coexistence or competitive exclusion.

## Results

### Comparing Logistic, Gompertz, and von Bertalanffy growth models

**Fig 1:**
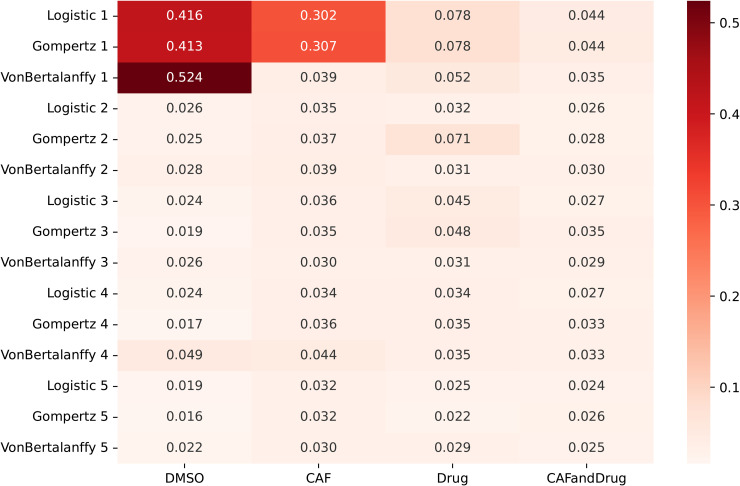
Chi-square error for the two-population model fits. Heatmap of the median of error values for the proposed fifteen models in Table 1 and S1 Appendix tables that account for Logistic, Gompertz, and von Bertalanffy growth models with different numbers of unknown parameters. The Logistic 1, Gompertz 1, and Von Bertalanffy 1 models have ρ, *K*, and λ as unknown parameters. The maximum number of unknown parameters in these models is five. Different growth models with more than three parameters yield insignificant variations in error. This means assuming more than four parameters leads to overfitting.

Our analysis demonstrates that fitting the two-population Logistic, Gompertz, and von Bertalanffy models results in satisfactory fits for all models with at least three fitted parameters. We checked AIC and error distributions for different environments and the number of unknown parameters to find which growth model is better. Still, the superiority of each growth model has no consistency. This can be due to the high correlation among the parameters and similarities of the models. The median error of the 15 models fit is illustrated in Fig 1. As seen in this figure, the median error is large when we have only two unknown parameters, such as in the model referred to as “Logistic 1” in Table 1. However, the difference between the error values for the rest of the models are very small, as presented in Fig 1. Comparisons based on the AIC align with the conclusions drawn from the error metric, as illustrated in S2 Appendix.

We note that allowing all the parameters to vary independently for each well results in overfitting, shown by minimal variations in error values as the number of parameters increases. All three models provided excellent fits to the data. Allowing all five parameters to vary independently for each well results in overfitting, shown by minimal variations in error values as the number of parameters increases Fig 1.

There is insufficient variation in goodness of fit metrics for determining the best growth model. To further explore the best fit growth model we used ANCOVA to test for non-linearities in density dependence by testing for effects of population size and population size squared on estimates of per capita growth rate (S5 Appendix). We ran a separate ANCOVA for all four combinations of DMSO versus CAF and cell type grown in monoculture. Logistic growth predicts a linear decline in per capita growth rate with cell number while Gompertz and van Bertalanffy predict a non-linear effect with a positive coefficient for the squared term. Regardless of cell type, in the presence of CAFs the squared term was not significant while under DMSO it was both positive and significant though very small (S5 Appendix). Although variations of von Bertalanffy with different exponents exist, we consider the classic $\frac{1}{3}$ exponent as this has been previously used to fit cancer populations [40]. In all cases per capita growth rate does decline with cell density as expected.

In investigating competition between the two cell types (sensitive and resistant), we did not want to use different growth models for CAFs and DMSO. Therefore, we chose the logistic growth model as our preferred model, as it assumes no non-linearities regarding density-dependence growth rates. Furthermore, logistic-growth extends easily into the Lotka-Volterra competition equations for estimating competition coefficients, and for comparing our different models for the effects of therapy on the sensitive cells.

### Determining drug efficacy term

**Fig 2:**
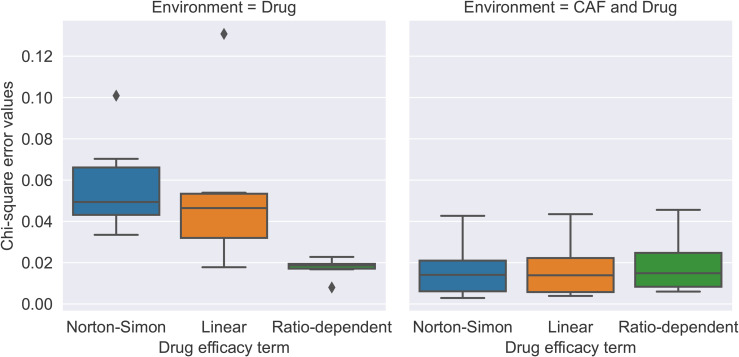
Chi-square error values for Norton-Simon, linear, and ratio-dependent drug efficacy (Eqs 5, 6, 7) in the presence of drug. The ratio-dependent drug efficacy model leads to smaller errors compared to Norton-Simon and linear drug effects in environment with the drug present.

We analyzed three treatment effects: Norton-Simon, linear, and ratio-dependent drug efficacies, introduced in Eqs 5, 6, and 7, respectively. Among these three models, ratio-dependent drug efficacy better fit the data and led to smaller chi-square errors, as observed in Fig 2. Furthermore, ratio-dependent drug efficacy aligns with the accepted biological understanding of population growth in relation to resource scarcity, whereas the Norton-Simon model does not. Our model uses λ>1 when the drug is administered, since the drug is administered in a constant dose [25]. Therefore, in the Norton-Simon model, for any λ>1, if the population exceeds the carrying capacity *K*, the population derivative $\dot{S}$ becomes positive and can increase indefinitely. Furthermore, for λ<1, the model is not representative of a decreasing population, indicating that this model does not accurately reflect the results of this experiment, where certain wells exhibit population decline.

### Parameter variations across cell types and CAFs presence

**Fig 3:**
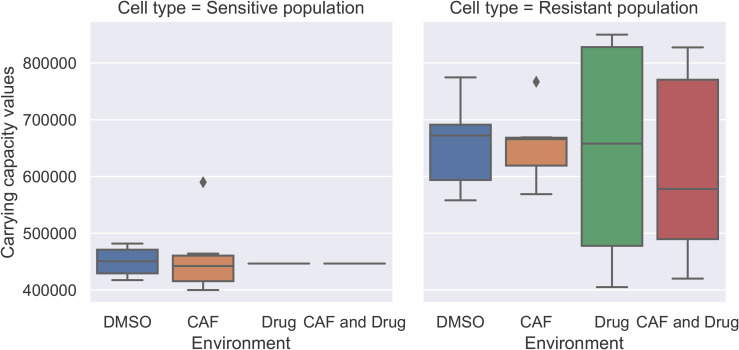
Carrying capacities (*K*_1_ and *K*_2_) in all environments. The boxplots of carrying capacity for sensitive and resistant populations illustrate the outcomes in different wells of the monotypic cultures. The carrying capacity of sensitive cells in the presence of the drug is fixed at the median of those parameters in DMSO and CAF environments. The cell type significantly affects carrying capacity, while the presence or absence of CAFs and drug results in minimal and non-significant variation.

**Fig 4:**
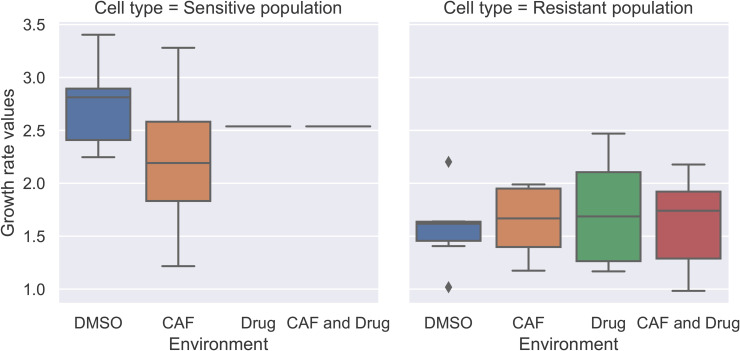
Growth rate parameters ($\rho_{1}$ and $\rho_{2}$) in all environments. The boxplots of growth rates for sensitive and resistant populations illustrate outcomes in different wells of the monotypic cultures. The growth rate of sensitive cells in the presence of the drug is fixed at the median of those parameters in DMSO and CAF environments. The cell types had significantly different growth rates, while the presence or absence of CAFs and drug resulted in minimal and non-significant variation.

We used logistic growth and ratio-dependent treatment effects to build the co-culture model. First, we fitted the sensitive and resistant cell population in wells where only one cell type is present (monocultures) in DMSO and CAF environments. We found estimates for growth rates of sensitive and resistant cells ($\rho_{1}$ and $\rho_{2}$) and their carrying capacities (*K*_1_ and *K*_2_). We observed that the growth rate and carrying capacity values of sensitive and resistant cells were not influenced by the presence and absence of CAFs when the drug was not present (Figs 3, 4).

Then, we fixed the sensitive population’s growth rate and carrying capacity parameters to the median values in the DMSO and CAF environments to estimate the drug efficacy parameter (λ) in environments with Alectinib present. Two-way ANOVA results supported our decision to set the sensitive population’s growth rate and carrying capacity to the median, indicating that population type significantly affected these parameters, whereas CAF presence did not (See details in S3 Appendix).

We do not expect the presence of therapy to impact the growth dynamics of the sensitive cell type in monoculture. To verify this assumption, we re-estimated the carrying capacity and growth rate parameters in environments containing only the drug and in those with both CAFs and the drug. The boxplots of carrying capacity for sensitive and resistant populations in Fig 3 demonstrate that population type has a greater influence on the carrying capacity parameter than the presence or absence of CAFs and drug. The lines shown in environments where the drug is present for sensitive cell population correspond to boxplots for fixed values. The variation in carrying capacity estimates of the resistant population in the presence of the drug is significantly higher than in other environments, perhaps due to more variation in the initial population in those wells. Although the initial ratio of sensitive and resistant populations is fixed at one and zero, respectively, the initial population size varies in different wells.

Fig 4 illustrates the boxplots of growth rate values. The growth rate estimates for both cancer cell types were not influenced by the environment. We fixed the growth rate of the sensitive population in DMSO and CAF environments and estimated the drug efficacy parameter (λ) in environments with Alectinib.

**Fig 5:**
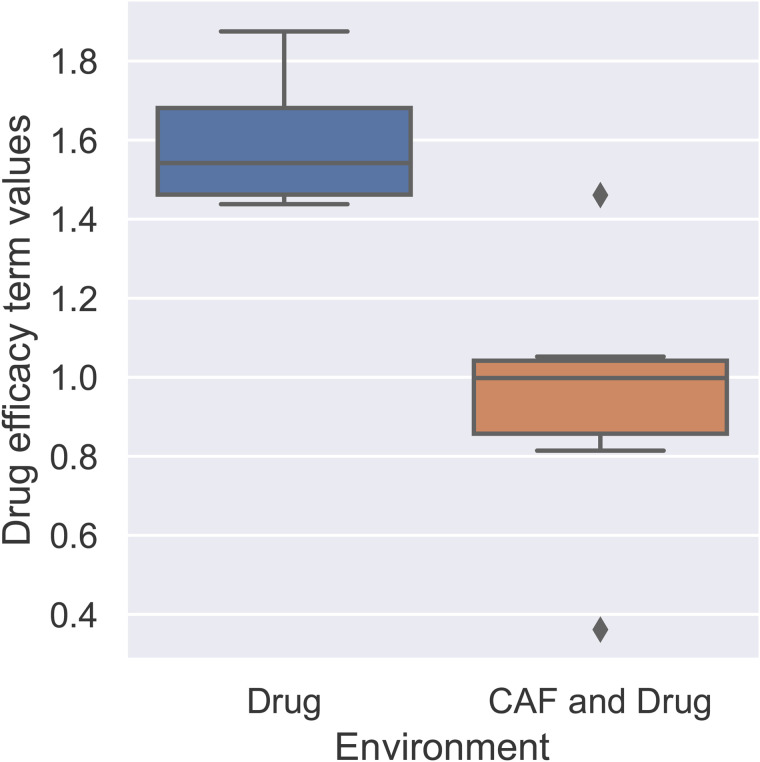
Drug efficacy (λ) in environments where drug is present. The boxplots of the drug efficacy parameter for sensitive populations illustrate the fit outcomes in different wells of monotypic culture. The presence of CAFs decreases drug efficacy.

The boxplots for drug efficacy (λ), illustrated in Fig 5, show that CAFs significantly decrease the effect of the drug on the sensitive cells’ population.

We used the median of ($\rho_{1},K_{1},\rho_{2},K_{2}$) parameters in all environments estimated from the monotypic cultures. However, considering that the presence of CAFs significantly influences the drug efficacy term (λ), we used the median of this term in the presence and absence of CAFs separately. Using the median of all other parameters, we estimated the competition coefficients ($\alpha_{SR}$ and $\alpha_{RS}$) in wells where both populations were present with different initial ratios.

The consideration of carrying capacity, growth rate, and drug effect as varying parameters resulted in overfitting and increased the correlation among the various parameters. Utilizing the estimates derived from the monocultures to construct the two-population model addressed these challenges.

**Fig 6:**
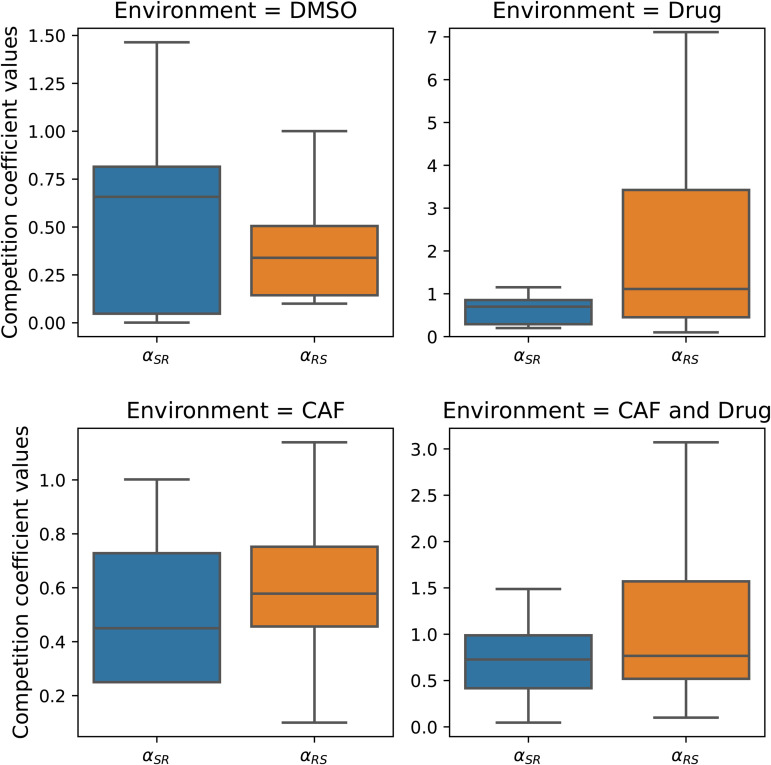
Competition coefficients ($\alpha_{SR}$ and $\alpha_{RS}$) derived for ratio-dependent drug effects and a logistic model where all parameters but competition coefficients were fixed. The presence of the drug leads to a large increase in the competition coefficient $\alpha_{RS}$. Note that the y-axis scale is different in the presence of the drug, and $\alpha_{RS}$ has values larger than 1.

From the boxplots of the competition coefficients derived from six different initial ratios in four environments, illustrated in Fig 6, we see that the presence of the drug increased the effect of sensitive cells on resistant ones through the competition coefficient $\alpha_{RS}$.

We used two two-way ANOVAs to test for the effects of drug and CAFs presence/absence on the two competition coefficients: $\alpha_{RS}$ and $\alpha_{SR}$. Both of these coefficients increased in the presence of drugs ($\alpha_{RS}=0.576$ and 2.144 in the absence and presence of drug, respectively, *F*_1,140_ = 17.64, *p* < 0.001; $\alpha_{SR}=0.519$ and 0.961 in the absence and presence of drug, respectively, *F*_1,140_ = 7.73, *p* < 0.01). There was no effect of CAFs or the interaction of CAFs and drug on either competition coefficient (See S4 Appendix for details). Furthermore, using a paired t-test comparing competition coefficients per well, there was no significant differences in the absence of drug (*t*_71_ = 0.75, *p* ≈ 0.45) but in the presence of drug $\alpha_{RS}$ was significantly greater than $\alpha_{SR}$ (*t*_71_ = 3.92, *p* < 0.001).

### Environmental influence on competition outcomes between cell types

**Fig 7:**
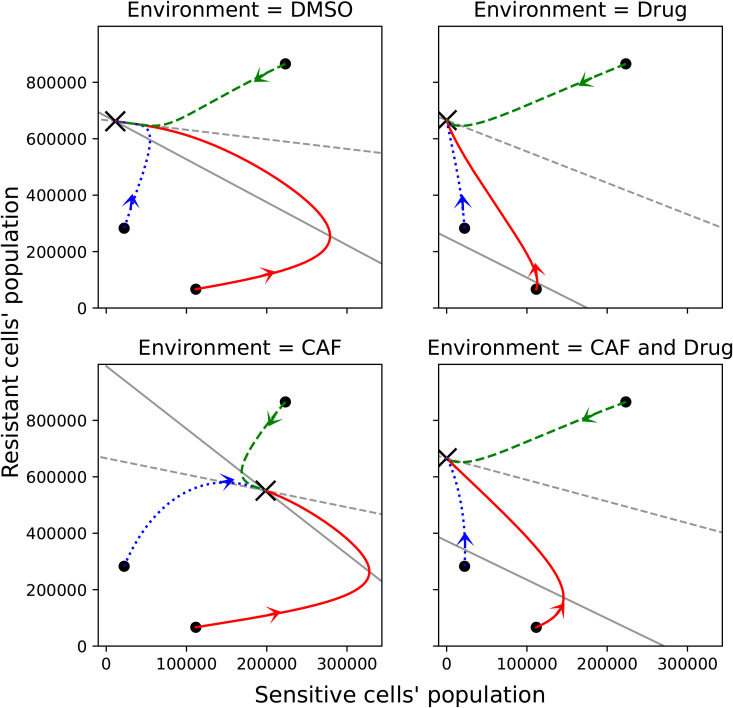
Part of phase plane of the model presented in Eq 7 using the median of the parameters derived from fitting the co-culture data in all environments. The × shows the stable equilibrium in each environment. The equilibrium in the CAF environment shows coexistence of resistant and sensitive cells. The equilibrium in DMSO shows the dominance of resistant cells. The equilibrium in environments where the drug is present shows that sensitive cells become extinct. The trajectories of the model, starting from three randomly chosen initial conditions, are illustrated in solid red, dashed green, and dotted blue lines. The solid grey line shows ($\frac{\dot{S}(t)}{S(t)}=0$) and the dashed grey line shows ($\frac{\dot{R}(t)}{R(t)}=0$), find more details in S6 Appendix.

Considering the median of competition coefficients derived from the co-culture experiment, we examine the outcomes of the competition between sensitive and resistant cell types in different environments by analyzing the Jacobian of the model (Eq 7) and its trajectories. We illustrate the trajectories from three different initial conditions in Fig 7. In the CAF environment, the model predicts coexistence of the two cell types. In the DMSO environment, the stable equilibrium occurs where the number of sensitive cells is much lower than that of resistant cells. In environments with the drug present, the resistant cells outcompete the sensitive ones.

The $\rho_{1}$, $\rho_{2}$, $K_{1}$, and $K_{2}$ parameters are considered to be the same in all four environments. Therefore, the different equilibrium properties in CAF compared to those at DMSO is the result of the increase in $\alpha_{RS}$ and decrease in $\alpha_{SR}$. The distribution of $\alpha_{SR}$ is not significantly different in DMSO and CAF. However, the median has a large difference, which strongly influences the equilibrium outcome. The presence of the drug decreases the number of sensitive cells and, through that, changes the equilibrium outcome. For more details on how the change in parameters λ and $\alpha_{SR}$ affects the possibility of coexistence, see S6 Appendix.

As illustrated in Fig 7, the intersection of the solid grey line with the vertical axis (the line S=0) where sensitive cells do not exist anymore marks a point that changes significantly with environmental variation. Specifically, in our models, this point varies with changes in the λ and $\alpha_{SR}$ parameter values, as shown in S6 Appendix. Furthermore, when the drug is absent, the λC(t) term becomes zero, causing the model’s nullclines to change. Notably, small $\alpha_{SR}$ leads to the differences we observe in Fig 7 regarding the existence of the mixed equilibria where cells coexist in the presence of CAF. In addition, the presence of CAF alters the chances for coexistence by decreasing the λ and $\alpha_{SR}$ parameter values. Even though in our model the presence of CAF and drug in the environment does not lead to coexistence, it raises the question of whether, if more CAF or less drug were present, λ and $\alpha_{SR}$ would be sufficiently small to result in coexistence. $\alpha_{RS}$ changes the slope of the line and therefore changes the intersection of the two nullclines.

## Discussion

Evolutionary game theoretic models can capture cancer eco-evolutionary dynamics and assist in optimizing its treatment [10,16,39,40]. In this paper, we analyzed data from experiments between two cancer cell lines from [25], also analyzed in [41]. We found that the two-population Lotka-Volterra competition model, which incorporates asymmetric competition and ratio-dependent drug efficacy, successfully captured the observed dynamics and offered novel mechanistic insight into how sensitive and resistant NSCLC cells interact under varying microenvironmental conditions. This ratio-dependent drug effect suggests that treatment effectiveness may depend not only on absolute tumour burden, but also on the relative composition of sensitive and resistant cells, a dynamic feature that can be leveraged in evolutionary therapy design.

Kaznatcheev et al. and Soboleva et al. fitted this data using replicator dynamics and a two-population Gompertz model with Norton-Simon death rate, respectively [25,41]. They both concluded that these models adequately capture the dynamics of the cancer cells studied. Both studies also reported that Alectinib inhibits the growth of sensitive cells. They also concluded that Alectinib does not affect the growth of resistant cells in monoculture. We confirm these results with our best-fit model as well. Our results show that the drug amplifies inter-cell type competition relative to intra-cell type. In particular, the sensitive cells now have a greater effect on the resistant cells than the sensitive cells have on themselves ($\alpha_{RS}=2.144$). By way of speculation, the extremely high $\alpha_{RS}$ could be a byproduct of the phenotypic mitigative response of the sensitive cells to the drug. They may greatly increase their metabolism, perhaps through upregulation of ABCC11 efflux pumps or upregulation of ALK proteins [42], thus amplifying resource depletion or metabolite production. The reduction in drug efficacy on sensitive cells in the presence of CAFs matches studies of stromal protection where CAFs and other stromal cells may confer partial resistance by detoxifying the drug or supplying mitigating factors to the cancer cells [43,44].

In co-culture, Kaznatcheev et al. concluded that cancer-associated fibroblasts (CAFs) enhanced the growth of sensitive cells [25]. We observed that CAFs both decreased resistant cells’ ability to outcompete the sensitive ones and increased the ability of the sensitive to persist in the population. This emerges from both density and frequency dependent effects. Resistant cells have the higher carrying capacity while the sensitive cells have a more favorable competition coefficient ($\alpha_{RS}>\alpha_{SR}$). Furthermore, we observed that CAFs decreased the effect of the drug through the drug efficacy parameter. We believe that if these observations are confirmed in future studies, they can help in designing novel therapies aimed at reducing cancer cells’ competitiveness, for example, by targeting the CAFs concentrations [45–47]. This aligns with growing interest in targeting CAF-mediated signalling to modulate tumour evolution and therapeutic response [45,46]. Immunotherapy may be the best type of treatment for this purpose [48,49].

Kaznatcheev et al. observed that resistant cells tend to have higher growth rates than sensitive cells even without the treatment targeting the sensitive cells [25]. Soboleva et al. did not confirm this conclusion [41]. Through our analysis, we found that resistant cells have a larger carrying capacity compared to sensitive ones, rather than having larger intrinsic growth rates. This distinction is critical for designing therapies that constrain tumour expansion without necessarily suppressing proliferation, an approach already shown to be effective in prostate cancer [16,50] and potentially translatable to NSCLC. This nuance could not be observed with the simpler models of Kaznatcheev et al. and Soboleva et al. As the first evolutionary therapies in prostate cancer targeted the carrying capacity of cancer cells rather than their growth rate [50], this suggests opportunities for optimizing treatment of non-small cell lung cancer as well.

Kaznatcheev et al. concluded that in the environment where only CAFs are present, the sensitive and resistant cells can coexist while in all other environments, the resistant cancer cell population will outcompete the sensitive population [25]. We also conclude that coexistence of the two populations is possible when only CAFs are present in the environment. In contrast, in environments where the drug is present, it leads to a fully resistant, stable equilibrium point. However, our model also predicts the coexistence of sensitive and resistant cells in DMSO, albeit with low abundances of sensitive cells. Kaznatcheev et al. concluded that resistant cells will outcompete sensitive cells under DMSO. Furthermore, the drug efficacy parameter λ is smaller in the presence of both CAFs and the drug compared to the environment where only the drug is present. We also confirm the positive effect of cancer-associated fibroblasts on the sensitive cells relative to the resistant cells.

Our model considers two types of cancer cells, one resistant to the Alectinib drug and one sensitive to it, that are well-mixed in the environment. While our model assumes a well-mixed population, this may not fully reflect spatial dynamics present in the experimental setup. If the two populations are not well-mixed, spatially explicit models may be more suitable to model their dynamics [51]. We could use agent-based, partial-differential equation, or other types of spatial models to address this issue [19,52,53]. Furthermore, we did not consider the resistance level of resistant cells as an evolving trait [14,54,55]. This aspect could be an interesting aspect for future research, as the resistant cells may evolve in the course of the experiments.

We estimated how the presence of CAFs and the Alectinib drug affect the model parameters and, through that, the outcome of competition between sensitive and resistant cells. We presented how the presence of CAFs results in the coexistence of sensitive and resistant cells. Reaching such an equilibrium may be possible through dose adjustment in response to the anticipated tumor burden [14,55–57].

Experiments are usually not performed to analyze both steady state behavior of the studied system and the transient dynamics leading towards this behavior. Even if that is the case, the number of replicates is often limited due to practical constraints. Here, we utilized our prior knowledge of the cells’ behavior and estimated model parameters for analyzing the steady-state behavior of cancer cells under different conditions.

The model we have found to be the most appropriate for capturing the dynamics of NSCLC within these experiments could form the basis for model-informed treatment. Of interest is treatment optimization within this model, to explore and compare treatment strategies to improve patients’ quality of life and survival. This aligns with the systems perspective emphasized by Soboleva et al. [58], where interdisciplinary models inform adaptive treatment strategies aimed at improving patient outcomes. As cancer therapy moves toward dynamic, model-informed approaches, frameworks like ours will be essential for translating eco-evolutionary insights into clinically actionable strategies.

## Supporting information

S1 Appendix General form of two-population models with Gompertz and von Bertalanffy growth. (PDF)

S2 Appendix AIC goodness of fit measure. (PDF)

S3 Appendix Details of two-way ANOVA Results (F-statistics and p-values) for Growth Rate and Carrying Capacity parameters. (PDF)

S4 Appendix Details of two-way ANOVA Results (F-statistics and p-values) on competition coefficient values. (PDF)

S5 Appendix ANCOVA test on relative growth. (PDF)

S6 Appendix Nullcline of the population model. (PDF)
