# Supplementary material for: Which evolutionary game-theoretic model best captures NSCLC dynamics?
Source: PLoS One. 2026 Jun 1;21(6):e0347657. doi: 10.1371/journal.pone.0347657 (PMC13225666; doi:10.1371/journal.pone.0347657)
Supplement: S1 Appendix — (PDF) [file pone.0347657.s001.pdf]

S1 Appendix. General form of two-population models with Gompertz and von Bertalanffy growth The general form of two-population models with Gompertz and von Bertalanffy growth and Norton-Simon drug effect is presented in Tables 1, 2.

Table 1. Models based on Gompertz growth

Gompertz 1	$\dot{S}(t) = \rho \ln(\frac{K}{S(t)+R(t)})(1-\lambda C(t))S(t)$ $\dot{R}(t) = \rho \ln(\frac{K}{S(t)+R(t)})R(t).$
Gompertz 2	$\dot{S}(t) = \rho_1 \ln(\frac{K}{S(t)+R(t)})(1-\lambda C(t))S(t)$ $\dot{R}(t) = \rho_2 \ln(\frac{K}{S(t)+R(t)})R(t).$
Gompertz 3	$\dot{S}(t) = \rho \ln(\frac{K}{S(t)+\alpha_{SR}R(t)})(1-\lambda C(t))S(t)$ $\dot{R}(t) = \rho \ln(\frac{K}{S(t)+R(t)})R(t).$
Gompertz 4	$\dot{S}(t) = \rho \ln(\frac{K}{S(t)+R(t)})(1-\lambda C(t))S(t)$ $\dot{R}(t) = \rho \ln(\frac{K}{\alpha_{RS}S(t)+R(t)})R(t).$
Gompertz 5	$\dot{S}(t) = \rho \ln(\frac{K}{S(t)+\alpha_{SR}R(t)})(1-\lambda C(t))S(t)$ $\dot{R}(t) = \rho \ln(\frac{K}{\alpha_{RS}S(t)+R(t)})R(t).$

Models inspired by Gompertz growth. Sensitive and resistant population growth follows the ODE model presented at each block. The unknown parameters in the Gompertz 1 model are ρ, K, and λ. Unknown parameters in Gompertz 2 are ρ_1, ρ_2, K, and λ. Unknown parameters in Gompertz 3 are ρ, K, α_{SR}, and λ. Unknown parameters in Gompertz 4 are ρ, K, α_{RS}, and λ. Unknown parameters in Gompertz 5 are ρ, K, α_{SR}, α_{RS}, and λ.

Table 2. Models based on von Bertalanffy growth

Von Bertalanffy 1	$\dot{S}(t) = \rho(1-\frac{\sqrt[3]{S+R}}{K})(1-\lambda C(t))S^{\frac{2}{3}}(t)$ $\dot{R}(t) = \rho(1-\frac{\sqrt[3]{S+R}}{K})R^{\frac{2}{3}}(t).$
Von Bertalanffy 2	$\dot{S}(t) = \rho_1(1-\frac{\sqrt[3]{S+R}}{K})(1-\lambda C(t))S^{\frac{2}{3}}(t)$ $\dot{R}(t) = \rho_2(1-\frac{\sqrt[3]{S+R}}{K})R^{\frac{2}{3}}(t).$
Von Bertalanffy 3	$\dot{S}(t) = \rho(1-\frac{\sqrt[3]{S+\alpha_{SR}R}}{K})(1-\lambda C(t))S^{\frac{2}{3}}(t)$ $\dot{R}(t) = \rho(1-\frac{\sqrt[3]{S+R}}{K})R^{\frac{2}{3}}(t).$
Von Bertalanffy 4	$\dot{S}(t) = \rho(1-\frac{\sqrt[3]{S+R}}{K})(1-\lambda C(t))S^{\frac{2}{3}}(t)$ $\dot{R}(t) = \rho(1-\frac{\sqrt[3]{\alpha_{RS}S+R}}{K})R^{\frac{2}{3}}(t).$
Von Bertalanffy 5	$\dot{S}(t) = \rho(1-\frac{\sqrt[3]{S+\alpha_{SR}R}}{K})(1-\lambda C(t))S^{\frac{2}{3}}(t)$ $\dot{R}(t) = \rho(1-\frac{\sqrt[3]{\alpha_{RS}S+R}}{K})R^{\frac{2}{3}}(t).$

Models inspired by von Bertalanffy growth. Sensitive and resistant population growth follows the ODE model presented at each block. The unknown parameters in the Von Bertalanffy 1 model are ρ, K, and λ. Unknown parameters in Von Bertalanffy 2 are ρ_1, ρ_2, K, and λ. Unknown parameters in Von Bertalanffy 3 are ρ, K, α_{SR}, and λ. Unknown parameters in Von Bertalanffy 4 are ρ, K, α_{RS}, and λ. Unknown parameters in Von Bertalanffy 5 are ρ, K, α_{SR}, α_{RS}, and λ.
