# Supplementary material for: Which evolutionary game-theoretic model best captures NSCLC dynamics?
Source: PLoS One. 2026 Jun 1;21(6):e0347657. doi: 10.1371/journal.pone.0347657 (PMC13225666; doi:10.1371/journal.pone.0347657)
Supplement: S2 Appendix — (PDF) [file pone.0347657.s002.pdf]

S2 Appendix. AIC goodness of fit measure Fig 8 illustrates the AIC value of the analyzed 15 models.

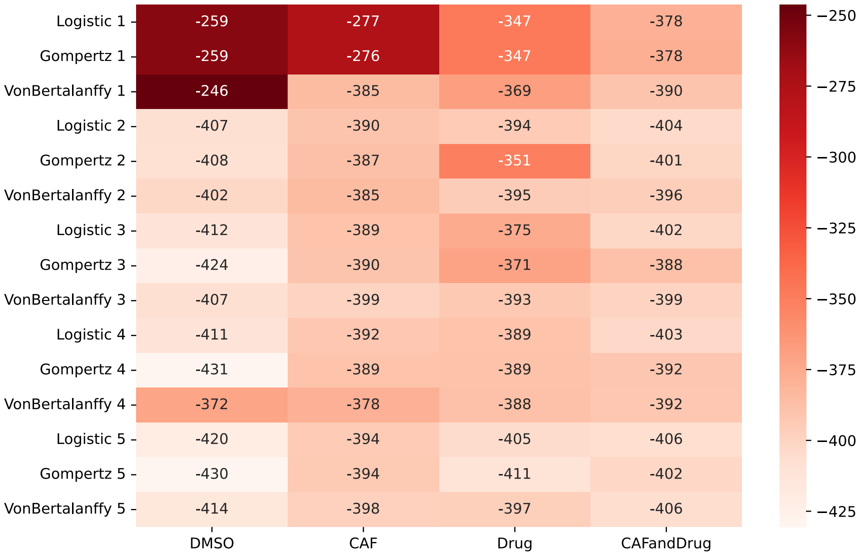

Fig 1. AIC result of the two-population model fits. Heatmap of the fit results for the proposed fifteen models. Models with more than three parameters fit the data well.
