# Supplementary material for: Which evolutionary game-theoretic model best captures NSCLC dynamics?
Source: PLoS One. 2026 Jun 1;21(6):e0347657. doi: 10.1371/journal.pone.0347657 (PMC13225666; doi:10.1371/journal.pone.0347657)
Supplement: S3 Appendix — (PDF) [file pone.0347657.s003.pdf]

S3 Appendix. Details of two-way ANOVA Results (F-statistics and p-values) for Growth Rate and Carrying Capacity parameters For the carrying capacity parameter, the ANOVA results were $F_{1,20} = 58.91, p < 0.001$ for population type; $F_{1,20} = 0.02, p = 0.88$ for CAF presence; and $F_{1,20} = 0.02, p = 0.87$ for the interaction effect (population type $\times$ CAF presence). For the growth rate parameter, the ANOVA results were $F_{1,20} = 18.50, p < 0.001$ for population type; $F_{1,20} = 2.1$, $p = 0.16$ for the interaction effect (population type $\times$ CAF presence).
