# Supplementary material for: Which evolutionary game-theoretic model best captures NSCLC dynamics?
Source: PLoS One. 2026 Jun 1;21(6):e0347657. doi: 10.1371/journal.pone.0347657 (PMC13225666; doi:10.1371/journal.pone.0347657)
Supplement: S4 Appendix — (PDF) [file pone.0347657.s004.pdf]

S4 Appendix. Details of two-way ANOVA Results (F-statistics and p-values) on competition coefficient values How the presence/absence of the drug and CAFs in the environment influences competition coefficients: For the a_{RS} parameter, the ANOVA results were $F_{1,140} = 17.64, p < 0.001$ for presence of drug; $F_{1,140} = 0.15, p = 0.69$ for CAF presence; and $F_{1,140} = 1.68, p = 0.19$ for the interaction effect. For the a_{SR} parameter, the ANOVA results were $F_{1,140} = 7.731$, $p = 0.006$ for presence of drug; $F_{1,140} = 0.71, p = 0.4$ for CAF presence; and $F_{1,140} = 1.09, p = 0.29$ for the interaction effect.
