# Supplementary material for: Which evolutionary game-theoretic model best captures NSCLC dynamics?
Source: PLoS One. 2026 Jun 1;21(6):e0347657. doi: 10.1371/journal.pone.0347657 (PMC13225666; doi:10.1371/journal.pone.0347657)
Supplement: S5 Appendix — (PDF) [file pone.0347657.s005.pdf]

S5 Appendix. ANCOVA test on relative growth To determine the growth dynamics, we analyze the relation between the population's relative growth ($\frac{1}{x}\frac{\Delta x}{\Delta t}$) with respect to the population (x). By determining the non-linearity order of the relationship, we can differentiate among logistic, Gompertz, and von Bertalanffy growth dynamics. The analysis is done for sensitive and resistant cells within monotypic cultures in DMSO and CAF environments. We use ANCOVA to identify the factors that significantly influence the relative growth of the cell population. The number of wells and cell population serve as primary experimental variables; cross-correlation addresses potential interactions; the intercept reflects baseline effects; and the second-order term allows the examination of non-linear relationships in growth behavior. In our first test, the relative growth is the dependent variable, the cell population is the covariate, and the number of wells is the group variable. We observe that the population's and intercept's effects are statistically significant in DMSO and CAF environments since the p-values of the population variable and intercept for sensitive and resistant cells are less than 0.001.

- In DMSO environment with only sensitive cells; for intercept, $F_{1,144} = 31.39, p < 0.001$; for sensitive population effect, $F_{1,144} = 16.94, p < 0.001$; for well number, $F_{5,144} = 0.56, p = 0.72$; for group-covariate correlation, $F_{5,144} = 0.38, p = 0.85$.
- In DMSO environment with only resistant cells; for intercept, $F_{1,144} = 39.46, p < 0.001$; for resistant population effect, $F_{1,144} = 24.04, p < 0.001$; for well number, $F_{5,144} = 0.73, p = 0.59$; for group-covariate correlation, $F_{5,144} = 0.63, p = 0.67$.
- In CAF environment with only sensitive cells; for intercept, $F_{1,144} = 23.50, p < 0.001$; for sensitive population effect, $F_{1,144} = 12.53, p < 0.001$; for well number, $F(5, 144) = 0.47, p = 0.79$; for group-covariate correlation, $F(5, 144) = 0.47, p = 0.79$.
- In CAF environment with only resistant cells; for intercept, $F_{1,144} = 15.59, p < 0.001$; for resistant population effect, $F_{1,144} = 4.69, p = 0.03$; for well number, $F_{5,144} = 2.12, p = 0.06$; for group-covariate correlation, $F_{5,144} = 1.63, p = 0.15$.

We repeated the test by removing the group-covariate correlation. The outcome that the group variable is insignificant stays as is. Furthermore, the significance of covariate variable for resistant population in CAF decreases from $p = 0.03$ to $p < 0.001$.

In the next step, we analyzed the significance of the second-order population term.

- In DMSO environment with only sensitive cells; for intercept, $F_{1,153} = 19.43, p < 0.001$; for sensitive population effect, $F_{1,153} = 7.87, p = 0.005$; for second order of population effect, $F_{1,153} = 4.21, p = 0.04$. ($Y_S = 0.035 - 1.09 \times 10^{-7} x_S + 9.41 \times 10^{-14} x_S^2$)
- In DMSO environment with only resistant cells; for intercept, $F_{1,153} = 35.76, p < 0.001$; for sensitive population effect, $F_{1,153} = 15.22, p < 0.001$; for second order of population effect, $F_{1,153} = 7.01, p = 0.008$. ($Y_R = 0.058 - 2.25 \times 10^{-7} x_R + 2.22 \times 10^{-13} x_R^2$)
- In CAF environment with only sensitive cells; for intercept, $F_{1,153} = 5.28, p = 0.02$; for sensitive population effect, $F_{1,153} = 0.77, p = 0.37$; for second order of population effect, $F_{1,153} = 0.04, p = 0.83$. ($Y_S = 0.021 - 3.88 \times 10^{-8} x_S + 1.04 \times 10^{-14} x_S^2$)

- In CAF environment with only resistant cells; for intercept, $F_{1,153} = 12.19, p < 0.001$; for resistant population effect, $F_{1,153} = 2.69, p = 0.10$; for second order of population effect, $F_{1,153} = 0.47, p = 0.49$. ($Y_R = 0.029 - 8.98 \times 10^{-8} x_R + 5.86 \times 10^{-14} x_R^2$)

The second-order term was not statistically significant for sensitive and resistant populations. We conclude that the Logistic model is more suitable here than Gompertz and von Bertalanffy since the relative growth is linearly related to the population.
