# Supplementary material for: Which evolutionary game-theoretic model best captures NSCLC dynamics?
Source: PLoS One. 2026 Jun 1;21(6):e0347657. doi: 10.1371/journal.pone.0347657 (PMC13225666; doi:10.1371/journal.pone.0347657)
Supplement: S6 Appendix — (PDF) [file pone.0347657.s006.pdf]

S6 Appendix. Nullcline of the population model $\dot{S} = 0$ result in two lines $S = 0$ and $\frac{\dot{S}}{S} = 0$ and $\dot{R} = 0$ result in two lines $R = 0$ and $\frac{\dot{R}}{R} = 0$. The intersection of $S = 0$ and $\frac{\dot{R}}{R} = 0$ results in equilibrium point P_1 at $(S, R) = (0, K_2)$. The intersection of $R = 0$ and $\frac{\dot{S}}{S} = 0$ results in equilibrium point P_2 at $(S, R) = (K_1(1 - \frac{\lambda}{\rho_1}), 0)$. The intersection of $\frac{\dot{S}}{S} = 0$ and $\frac{\dot{R}}{R} = 0$ results in a mixed equilibrium point, P_3, which might not exist for some parameter values. The value of P_4: $(S, R) = (\frac{K_2}{\alpha_{RS}}, 0)$ and P_5: $(S, R) = (0, \frac{K_1}{\alpha_{SR}}(1 - \frac{\lambda}{\rho_1}))$ could be higher and lower than P_2 and P_1, respectively, depending on parameter values. This determines whether the mixed equilibrium point exists or not. As illustrated in Fig 2, depending on α_{SR}, α_{RS}, and λ values, there can be cases where the mixed equilibrium located at P_3 does not exist. For our parameter values, P_4 is always larger than P_2. However, in the presence of the drug, λ and α_{SR} become larger. Due to this change, P_5 becomes smaller than P_1, which leads to disappearance of the mixed equilibrium points and extinction of sensitive cells. Furthermore, adding only CAFs to the DMSO environment leads to a decrease in α_{SR}, which causes P_5 to be larger than P_1, leading to a mixed equilibrium point with a large population of sensitive cells.

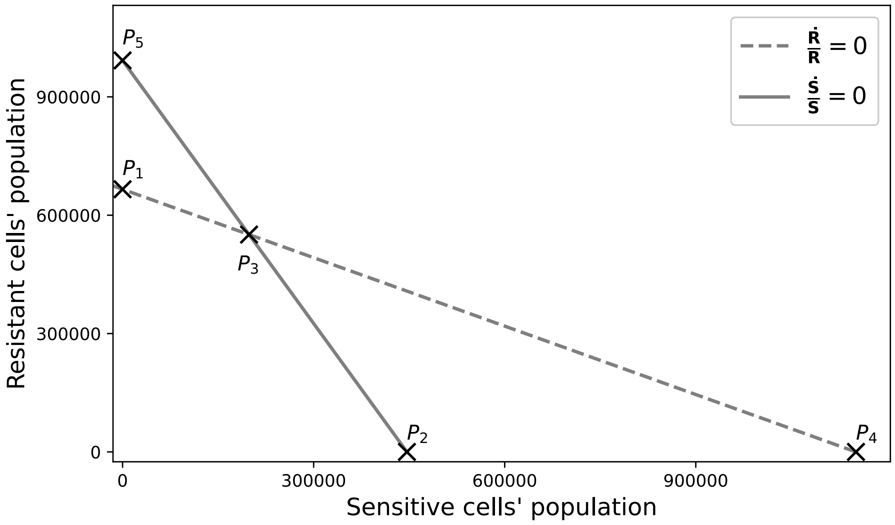

Fig 2. Intersection of nullclines of Eq 7. Setting the derivative of sensitive population to zero, $\dot{S} = 0$, results in two lines $S = 0$ and $\frac{\dot{S}}{S} = 0$. Setting the derivative of resistant population to zero, $\dot{R} = 0$, results in two lines $R = 0$ and $\frac{\dot{R}}{R} = 0$. P_1 point shows $(S, R) = (0, K_2)$; P_2: $(S, R) = (K_1(1 - \frac{\lambda}{\rho_1}), 0)$; P_3 shows the mixed equilibrium which might not exist for some parameter values; P_4: $(S, R) = (\frac{K_2}{\alpha_{RS}}, 0)$; P_5: $(S, R) = (0, \frac{K_1}{\alpha_{SR}}(1 - \frac{\lambda}{\rho_1}))$.
